# The inflammatory response in the regression of lumbar disc herniation

**DOI:** 10.1186/s13075-018-1743-4

**Published:** 2018-11-06

**Authors:** Carla Cunha, Ana J. Silva, Paulo Pereira, Rui Vaz, Raquel M. Gonçalves, Mário A. Barbosa

**Affiliations:** 1 i3S—Instituto de Investigação e Inovação em Saúde, Universidade do Porto, Rua Alfredo Allen 208, 4200-135 Porto, Portugal; 2 INEB—Instituto de Engenharia Biomédica, Universidade do Porto, Rua do Campo Alegre 823, 4150-180 Porto, Portugal; 3 Department of Neurosurgery, Centro Hospitalar São João, Porto, Portugal; 4 Department of Clinical Neurosciences and Mental Health, Faculty of Medicine, University of Porto, Porto, Portugal; 5 Neurosciences Center, CUF Porto Hospital, Porto, Portugal; 6 ICBAS—Instituto de Ciências Biomédicas Abel Salazar, Universidade do Porto, Rua Jorge Viterbo Ferreira 228, 4050-313 Porto, Portugal

**Keywords:** Low back pain, Spine, Intervertebral disc, Immunomodulation, Macrophages

## Abstract

Lumbar disc herniation (LDH) is highly associated with inflammation in the context of low back pain. Currently, inflammation is associated with adverse symptoms related to the stimulation of nerve fibers that may lead to pain. However, inflammation has also been indicated as the main factor responsible for LDH regression. This apparent controversy places inflammation as a good prognostic indicator of spontaneous regression of LDH. This review addresses the molecular and cellular mechanisms involved in LDH regression, including matrix remodeling and neovascularization, in the scope of the clinical decision on conservative versus surgical intervention. Based on the evidence, a special focus on the inflammatory response in the LDH context is given, particularly in the monocyte/macrophage role. The phenomenon of spontaneous regression of LDH, extensively reported in the literature, is therefore analyzed here under the perspective of the modulatory role of inflammation.

## Low back pain and lumbar disc herniation

Lumbar disc herniation (LDH) is a major contributor to low back pain and affects around 9% of all people worldwide, with a high associated economic burden and a tendency to increase as the population ages [1]. LDH has been associated with disruption of the annulus fibrosus (AF), extrusion of the nucleus pulposus (NP), and stimulation of nerve fibers, leading to pain. However, more recently, Rajasekaran et al. [2] suggested that disc herniation is more commonly the result of endplate junction failure than AF rupture. Herniated discs are found in 30–40% of asymptomatic people by imaging diagnostic tools [3].

The current treatments for LDH, as well as for degenerative disc disease in general, can be divided into conservative versus surgical approaches and the decision on which approach to use is variable and patient–clinician dependent. Symptoms originated by LDH may disappear without any surgical treatment and in some of these patients this is accompanied by a reduction of the size of disc herniation in imaging studies. This phenomenon is known as spontaneous hernia regression, which may be partial or complete. Clearly, this evidence is indicative of the paramount need to identify the mechanisms behind LDH regression and to develop predictive methods for detection of this phenomenon in clinical practice.

## Clinical evidence of spontaneous LDH regression

**Fig. 1:**
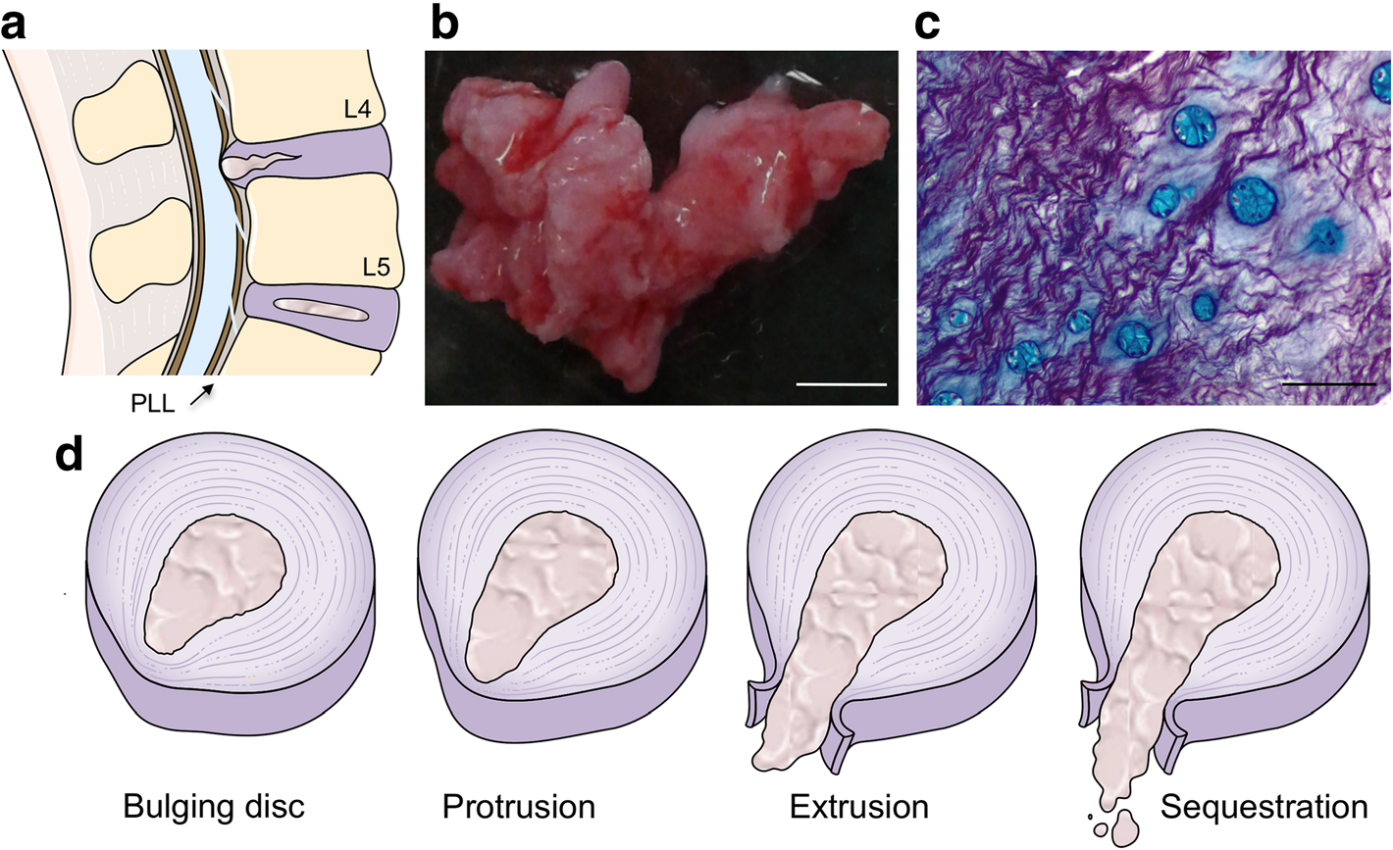
**a** Schematic representation of typical L4–L5 hernia, with compression and possible rupture of posterior longitudinal ligament (PLL). **b** Human LDH fragment, obtained from patient who underwent microdiscectomy after informed consent and ethics committee approval from Centro Hospitalar São João. **c** Histological staining of tissue collected in (b), showing cell clusters producing proteoglycans (Alcian blue) embedded in a collagen matrix (Picrosirius red). **d** LDH is currently divided into four subtypes, according to MRI, as bulging disc (mildest form), protrusion, extrusion, and sequestration, the severest form of LDH. Proteoglycan-rich nucleus pulposus in center is surrounded by collagen-rich concentric rings of annulus fibrosus. Scale bars: (**b**) 3 cm, (**c**) 100 μm. Image credits: (**a**, **d**) used elements from Servier Medical Art; (**b**, **c**) unpublished

Since the first report of spontaneous LDH regression [4] and of computed tomography (CT)-confirmed spontaneous LDH regression [5], documentation of this phenomenon has become broadly available in the literature. Imaging diagnostic tools such as magnetic resonance imaging (MRI) have a central role in confirming LDH regression and, although controversial for a significant number of years, it is now widely recognized that large-sized and sequestrated LDH tend to regress more than other LDH subtypes (Fig. 1). This regression may be partial or complete. The level most commonly affected by this phenomenon is L4–L5, which is also where LDH occurs more frequently [6]. The main hypothesis behind the initiation of spontaneous LDH regression has been described as the exposure of herniated disc material to the epidural vascular supply through the ruptured posterior longitudinal ligament (PLL) (Fig. 1).

MRI analyses that allow precise quantifications of the decrease in herniation volume have shown that spontaneous LDH regression is more related to the presence of transligamentous extension and not so much to the initial size of herniation. Ahn et al. analyzed longitudinally in time 36 symptomatic herniated lumbar discs and showed that 25 of them decreased in size and that the most frequent herniation types that reduced in size were sequestered herniations (average decreases of 17%, 48%, and 82% for the subligamentous, transligamentous, and sequestered herniation groups, respectively). These results suggest that the PLL rupture is more important than the initial size of the hernia [7]. In Takada et al.’s study [8], all cases of sequestrated discs were completely resolved after 9 months, whereas extruded discs were only completely resolved after 12 months. On the other hand, disc protrusions showed little to no signs of regression after 12 months, most probably because of the patients’ younger age and the abundance of collagen fibers and chondrocyte-like cells from the NP in these discs. The faster rate of radiographic resolution seen with sequestrated discs has been traditionally associated with dehydration and shrinkage, as the free fragment is no longer supplied with nutrients from the parent disc [9, 10]. Other authors postulate that sequestrated discs, unlike other LDH subtypes, trigger an inflammatory response characterized by neovascularization and immune cell-mediated degradation [11–13]. The subsequent increase in blood flow around free fragments explains why the periphery of disc sequestrations is enhanced with gadolinium (Gd) contrast, which is measured by MRI rim enhancement. In fact, Autio et al. [11] proposed that sequestrated discs with higher levels of Gd diethylenetriamine pentaacetic acid (Gd-DTPA) enhancement on MRI images served as predictors for their higher resorption rate. In this study, MRI of herniated patients was repeated throughout 12 months showing that a significant NP resorption occurred until 2 months after diagnosis and was more pronounced over the 1-year follow-up period. Higher resorption rates were associated with higher baseline scores of rim enhancement thickness, higher degree of herniated NP displacement in the Komori classification, and an age range of 41–50 years. The thickness of rim enhancement was a stronger determinant of spontaneous resorption than its extent. The extent of rim enhancement significantly correlated with the degree of disc displacement, being most pronounced in the case of sequesters.

### Clinical decision on conservative versus surgical intervention for LDH

In order to understand spontaneous LDH regression, its prognostic factors, and its predictive outcomes, large cohorts have been conducted, namely the Maine Lumbar Spine Study [14], the Spine Patient Outcomes Research Trial (SPORT) [15], and the Hague Spine Intervention Prognostic Study Group [16], each one enrolling hundreds of patients suffering from LDH. Interestingly, all of these studies indicate that early surgery achieves more rapid relief of LDH symptoms than conservative care but, in the long run, outcomes gradually become identical to conservative treatment. Many other systematic studies have consistently obtained similar results [17–20]. Buttermann [21] compared the LDH symptoms of patients after 6 weeks of conservative treatment (38 patients) with those who received epidural injections (20 patients) and found that both groups had similar outcomes, including the size of herniated NP.

Despite the evidence for spontaneous LDH regression, there is still much debate among clinicians concerning the efficacy of conservative vs nonconservative treatments. Initial management for patients with the sequestrated subtype of LDH may be conservative due to the higher likelihood and faster rate of resolution in comparison to the other LDH subtypes (reviewed in [6, 22]). Patients with intractable pain, neurological deficit, or bowel or bladder dysfunction, among other associated factors, remain candidates for earlier surgical intervention. However, it is still hard to predict which patients are more likely to benefit from conservative treatment and have higher probability of spontaneous regression of the disc herniation. It is therefore remarkably challenging to decide which patients to submit to surgery. Future large, prospective, randomized trials are required to better determine a set of specific clinical criteria, such as those defined by Chiu et al. [22], that may predict the surgery outcome. On a more mechanistic approach, finding molecular markers, possibly noninvasive ones, such as systemic markers, to predict the outcome of LDH would have a major impact on the clinical decision. These approaches are only starting to emerge, such as the study by Elkan et al. [23] in which high plasminogen activator inhibitor 1, a marker of fibrinolysis, analyzed in blood samples of patients with LDH, was fairly consistently associated with poor LDH surgery outcome.

## Mechanisms behind LDH spontaneous regression

As already described, the spontaneous regression of IVD herniated tissue is well documented clinically, but the underlying mechanisms remain unclear. To the best of our knowledge, three theories have been proposed to explain the resorption of herniated material. The first theory proposes that the herniated disc fragment reduces in size due to gradual dehydration and shrinkage, which may explain the decrease of signal intensity of the disc in the follow-up MRI studies [9, 10]. The second hypothesis suggests that tension applied by the PLL leads to retraction of the herniated disc fragment back into the IVD space. This mechanism may explain the cases where the herniated disc has an intact AF, but not the cases with completely extruded or migrated disc fragments [5]. Onel et al. [24] showed that under a static traction load of 45 kg, the herniated tissue retracted in 21 out of 30 LDH patients, while in two of them the herniated tissue has actually increased. The third theory, the most extensively studied with preclinical and clinical evidence to support it, is the gradual hernia resorption through enzymatic degradation and phagocytosis induced by an inflammatory reaction and neovascularization [11–13]. This inflammatory reaction is supposed to be triggered when the disc content extrudes into the epidural space and is then recognized as foreign. Depending on each individual clinical condition, it is possible that one specific mechanism or different combinations of the three may operate in spontaneous regression of the herniated disc tissue.

### The privileged immunity of IVD

The IVD is the largest avascular organ in the human body and considered an immune-privileged site [25]. The NP appears to be particularly isolated from the immune system of the host, given its position between two cartilaginous endplates and inside the dense collagen fibrous structure of the AF. Additionally to this physiological barrier, the IVD cells also actively resist invasion by immune cells, due to the Fas ligand (FasL) expression, which is characteristic of immune-privileged sites [26]. FasL belongs to the tumor necrosis factor (TNF) family and is known to induce apoptosis by binding to its receptor, Fas. While Fas is expressed in a wide variety of cells, FasL expression is restricted to the surface of cytotoxic T cells, natural killer (NK) cells, tumor cells, and stromal cells of some immune-privileged sites. In immune-privileged sites, FasL of stromal cells binds to Fas receptor expressed on immune cells and infiltrating cells. This ligand–receptor binding induces apoptosis of the infiltrating immune cells, maintaining the immune-privileged condition of the tissue [27]. The immune-privileged environment of the NP is the pillar of the theory of the inflammatory reaction behind LDH resorption. This theory proposes that the extrusion of the NP tissue into the epidural space evokes an autoimmune reaction that leads to the infiltration of immune cells which will interact with IVD cells and secrete a variety of molecules initiating the hernia resorption process [11, 12, 28].

### Macrophages as key players in LDH regression

Macrophages are indicated as the most important immune players in the resorption process of herniated discs. Numerous studies have found by immunohistochemistry the presence of macrophages in herniated IVD tissue specimens [29–32]. These cells have the capability to actively phagocyte the herniated tissue and process it in their lysosomes filled with collagen-degrading enzymes. Macrophages also secrete lysosomal enzymes by exocytosis, which break down intercellular substances such as the disc matrix components proteoglycans and collagens [12, 33]. Furthermore, phagocytic activity of macrophages was observed in surgically removed samples of herniated NP through electron microscopy [12] and these immune cells are known to express scavenger receptors, such as CD36, which have been characterized as the main responsible molecules for the phagocytosis of apoptotic cells, highlighting the potential role of macrophages in hernia resorption [34]. Interestingly, in LDH histological samples, macrophage phagocytosis was observed more often in sequestration subtype LDH than subligamentous ones [35], in accordance with the clinical evidence showing that sequestered hernias are more likely to regress. Ikeda et al. [29] also observed more frequently the infiltration of macrophages and neovascularization along the margins of the transligamentous extruded disc material than in other subtypes of LDH.

#### Monocyte recruitment to IVD tissue

The exact mechanism by which monocytes are recruited to the IVD remains unclear, as reviewed previously [36]. However, it is known that the IVD endogenously includes inflammatory-like cells (i.e., cells with phagocytic capacity) and IVD cells are able to produce inflammatory mediators [36], which may themselves contribute to recruit other immune cells to the hernia site. In particular, monocyte chemoattractant protein (MCP)-1, a CC chemokine that contributes to the activation and recruitment of monocytes, has been extensively demonstrated to be produced by IVD cells [13, 37–40]. In particular, Yoshida et al. [13] developed a hernia model in which IVD cells produced proinflammatory cytokines as an initial response to disc herniation. These cytokines stimulate the production of MCP-1 by IVD cells, resulting in macrophage infiltration in herniated discs. The infiltrating monocyte-derived macrophages also produce MCP-1, increasing the monocyte recruitment to the IVD [13]. Apart from macrophages, plasmacytoid dendritic cells have also been shown to be involved in LDH resorption [41]. Surgical material from transligamentous and subligamentous sequestrations was analyzed by flow cytometry and plasmacytoid dendritic cells were found to predominate over macrophages on transligamentous sequestrations, indicating that these cells may be involved in the initiation of the immune response [41].

#### Inflammatory cascades in LDH-implicating macrophages

Numerous studies analyzed immune mediators in LDH and especially implicating macrophages in LDH regression (Table 1). Shamji et al. [32] showed high expression levels of macrophage products like IL-4, IL-6, IL-12, and interferon gamma (IFN-γ) in herniated disc tissue. Other studies demonstrated that IVD tissue is also capable of spontaneously producing other molecules, such as the chemokines IL-8 and MCP-1, the main functions of which are chemotaxis of macrophages and angiogenesis [37]. Furthermore, Kang et al. [42] demonstrated that herniated discs release high levels of matrix metalloproteinases (MMPs), nitric oxide (NO), IL-6, and prostaglandin E2 (PGE2) and this production increases when the discs are stimulated with IL-1β, evidencing that IVD cells are biologically responsive to exogenous stimuli. Regarding IL-6, it has already been demonstrated in vitro that its production is induced when IVDs and macrophages are cocultured [43]. More recently, Takada et al. [44] showed that coculturing IVDs and macrophages upregulated IL-8, PGE2, and cyclooxygenase 2 (COX-2). In both studies, the mentioned biochemical mediators are mainly produced by macrophages. The latter study also showed that rat IVD autografts induced extensive macrophage infiltration in vivo, increasing the mRNA levels of TNF-α, IL-6, IL-8, and COX-2. TNF-α is required for IL-6 and PGE2 production, but not for IL-8 production, during IVD–macrophage interaction. Neutralization of TNF-α and IL-8 may be a valuable therapy for pain related with LDH [44]. Other studies have also analyzed the interaction between macrophages and IVD tissue using cocultures between macrophages and chondrocytes or whole IVD. Haro et al. demonstrated that the production of both MMP-3 and MMP-7 was strongly upregulated in IVD cell/macrophage coculture, MMP-3 being produced by both chondrocytes and macrophages while MMP-7 is produced predominantly by macrophages. Moreover, the authors also revealed that disc-derived MMP-3 is required for the physical degradation of disc tissues and for macrophage infiltration, which ultimately leads to hernia resorption [45]. In other studies, the same group demonstrated that MMP-7 released by macrophages contributes to the process of herniated disc resorption through the release of soluble TNF-α [46, 47]. TNF-α is a potent inducer of many MMPs, such as MMP-3, and also of vascular endothelial growth factor (VEGF) that is implicated in the neovascularization of herniated discs [47, 48]. The crucial role of MMP-7 in the initiation of herniated disc resorption resulted in the development of a recombinant human MMP-7 intradiscal therapy, which is in phase I/II clinical trials in the United States. This therapy avoids the side effects associated with surgery, such as nerve tissue damage [49].

**Table 1:** Immune mediators implicating macrophages in LDH regression

Immune mediator	Sample	Species	Study
IL-6, NO, PGE2, MMP-3, MMP-2/MMP-9	Herniated IVD tissue	Human	Kang et al., 1997 [42]
IL-8, MCP-1	Herniated IVD tissue	Human	Burke et al., 2002 [37]
IL-4, IL-6, IL-12, IFN-γ	Herniated IVD tissue	Human	Shamji et al., 2010 [32]
MMP-3, MMP-7	Coculture of IVD and macrophages	Mouse	Haro et al., 2000 [45]
MMP-7, TNF-α	Coculture of IVD and macrophages	Mouse	Haro et al., 2000 [46]
MMP-3, MMP-7, TNF-α, VEGF	Coculture of IVD and macrophages	Mouse	Kato et al., 2004 [47]
IL-6	Coculture of IVD and macrophages	Rat	Takada et al., 2004 [43]
IL-8, PGE2, COX-2	Coculture of IVD and macrophages	Rat	Takada et al., 2012 [44]

*COX-2* cyclooxygenase 2, *IFN-γ* interferon gamma, *IL* interleukin, *IVD* intervertebral disc, *LDH* lumbar disc herniation, *MCP-1* monocyte chemoattractant protein-1, *MMP* matrix metalloproteinase, *NO* nitric oxide, *PGE2* prostaglandin E2, *TNF-α* tumor necrosis factor alpha, *VEGF* vascular endothelial growth factor

### Neovascularization in LDH resorption

Altogether, these studies provide evidence for a mechanism of LDH resorption associated with a cascade of inflammation, matrix remodeling, and angiogenesis. However, only few studies have specifically addressed neovascularization.

**Fig. 2:**
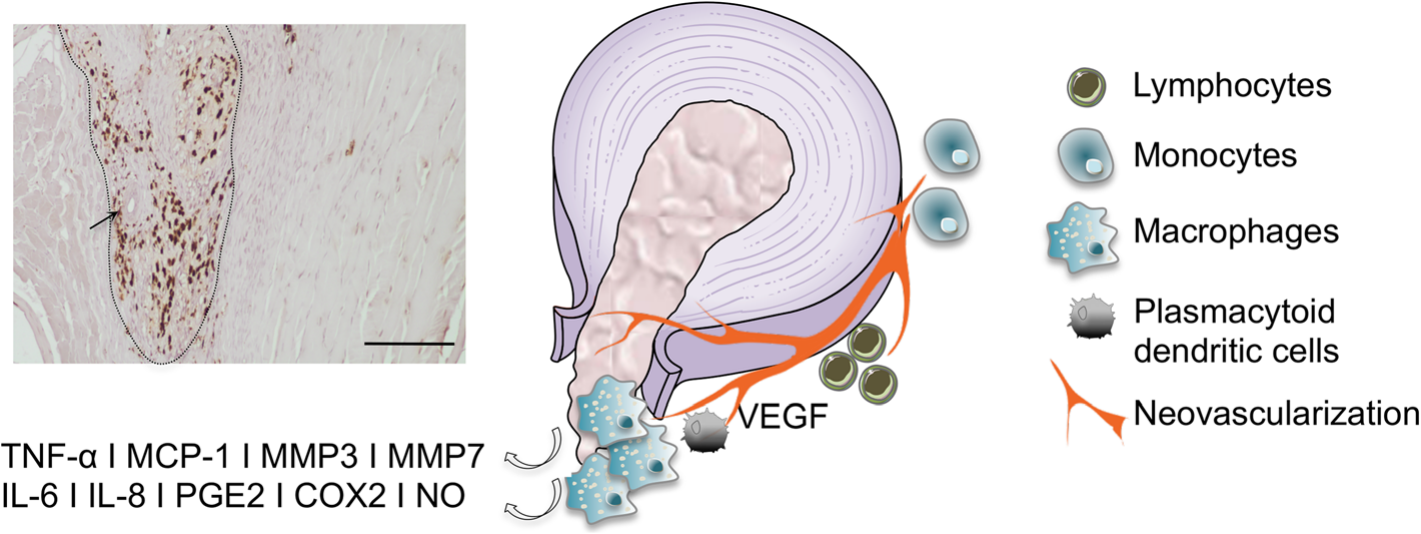
Representative proposed mechanism of LDH resorption. Both herniated IVD tissue and macrophages produce tumor necrosis factor alpha (TNF-α), monocyte chemoattractant protein (MCP)-1, matrix metalloproteinases (MMPs), interleukin (IL)-6, IL-8, prostaglandin E2 (PGE2), cyclooxygenase 2 (COX2), and nitric oxide (NO), which contribute to the inflammatory reaction and resorption of the herniated tissue. Vascular endothelial growth factor (VEGF) induces blood vessel ingrowth and neovascularization, which support immune cell mobilization to hernia site. Insert: in rat model of IVD herniation, CD68^+^ macrophages localized within hernia (delimitated by dashed line), which include a blood vessel (arrow). Scale bar: 100 μm. Image used elements from Servier Medical Art; insert: unpublished

Usually, few blood vessels exist in the mature IVD. However, proliferation of new vessels has already been demonstrated at the margin of the herniated tissue and this is thought to be a major determinant of spontaneous regression of LDH [11, 50]. Several molecular mediators have been suggested to be involved in the neovascularization of LDH, including TNF-α, VEGF, and basic fibroblast growth factor (bFGF). As mentioned earlier, TNF-α can promote the expression of VEGF, which plays an essential role in the formation of new blood vessels, and some studies confirmed the presence of VEGF and VEGF receptors in human LDH tissue [48, 51]. Also, the interaction between macrophages and disc tissue leads to generation of inflammatory cytokines, which are known to be involved in the induction of angiogenesis. Haro et al. observed an increase in the mRNA and protein levels of VEGF when macrophages were in contact with disc tissue comparatively to the expression levels when they were cultured alone. This enhancement of the VEGF levels was apparently mediated by a TNF-α-dependent pathway since this effect was abrogated by the use of a TNF-α neutralizing antibody [48]. Using MRI in humans, the presence of capillaries invading the hernia and monocyte-derived macrophages migrating out of these capillaries has been demonstrated [12]. Additionally, in an experimental model in rabbits, bFGF, a factor known to stimulate mitogenesis and chemotaxis of fibroblasts and capillary endothelial cells, as well as to stimulate angiogenesis, promoted the resorption of disc material. In this study, treatment with bFGF led to an increase in the number of newly formed vessels with a consequent higher infiltration of inflammatory cells (macrophages, lymphocytes, and fibroblasts), which contributed to the resorption process [52].

These studies reinforce the importance of the crosstalk between angiogenesis and inflammation, which ultimately leads to LDH regression.

### Contribution of other immune response mediators

An autoimmune reaction is far more complex than the mere macrophage recruitment and neovascularization so far described. Indeed, apart from macrophages, other immune cells are present in LDH (Fig. 2). The complete understanding of the immune response associated with LDH regression has benefited to a great extent from in-vivo animal models. More than in-vivo evidence for LDH resorption, already thoroughly clinically demonstrated, animal models have been employed to unravel the underlying mechanisms of IVD regression. We have shown spontaneous regression of LDH in a rat IVD lesion model [53]. In this work, IVD lesion was induced by either 21-G or 25-G needle puncture and we have found that the size of the hernia formed was proportional to the needle gauge used. In both cases, hernias significantly diminish in volume from 2 to 6 weeks post injury. Also, we found that the number of CD68^+^ macrophages within the hernia as well as cell apoptosis within the tissue were both proportional to the hernia volume. Using the same model, we further confirmed that the number of CD68^+^ macrophages in the hernia was proportional to its size and hypothesized that only a certain number of macrophages will be recruited and activated per area of hernia, keeping the tissue homeostasis. Moreover, we have found that the systemic transplantation of rat bone marrow MSC resulted in a significant reduction in the size of the hernias formed 2 weeks post lesion and that the number of B lymphocytes surrounding the hernia increased [54]. In another study, a rabbit herniation model was developed, consisting of the introduction of a needle up to penetrating the PLL and then physically compressing to extrude the NP to the epidural space [13]. The herniated discs spontaneously reduced in size gradually up to 12 weeks post surgery. Infiltrating cells, mainly composed of macrophages, were observed from day 3. Immunohistochemically, IVD cells in the herniated discs produced TNF-α and IL-1β on day 1, followed by MCP-1 on day 3.

Nevertheless, most of the current in-vivo LDH models are autotransplantation models in which IVDs are transplanted into subcutaneous or dorsal epidural spaces of the animals after laminectomy, with the scope of specifically analyzing the associated immune response. This is because it is still controversial whether the inflammatory response of the host is initiated simply by exposure to structural elements and compounds that are present in the disc cell membrane and matrix, by direct contact of the NP material with the immune system via an autoimmune response, or whether it is secondary to an autoimmune response. It has been shown previously that subcutaneously transplanted NP cell survival was reduced in association with an immunological reaction, by transplanting NP tissue into Lewis rats and into NOD mice, and that NK cells and macrophages were present around the outgrown NP tissues but no T cells were found [55]. Another study implanted autologous NP subcutaneously in pigs and activated T cells (CD4^+^ and CD8^+^) were found in the exudates in considerable number, as well as activated B cells expressing immunoglobulin kappa (Igκ). The results showed that NP attracts activated T and B cells [56].

What most of these studies show is that the immune system is able to respond to the intact healthy NP tissue. To analyze whether inflammation occurs in response to compounds secreted from viable cells in the NP or whether inflammation simply requires exposure to structural cell or matrix components, Rand et al. [57] used a mouse model in which the animal was exposed to disc tissue containing viable NP and AF cells, to disc tissue containing viable AF cells, or to disc tissue with no viable cells. The three tissue preparations were inserted into the right lower peritoneal cavity. The devitalized tissue was intended to assess a possible effect of chemical irritants that may be present in the disc and that may induce inflammation. Macrophage recruitment occurred over the course of 1, 2, and 7 days post injury and only after exposure to viable disc tissue but not after exposure to devitalized disc components [57], indicating that macrophage recruitment occurred only in response to cell cues.

Most of the evidence for the inflammatory reaction around spontaneous hernia regression has been collected from animal studies. Despite this, some studies mostly involving immunohistochemistry analysis of clinical samples have confirmed the results obtained in animal experimentation. The presence of T and B lymphocytes on isolated human herniated discs was further confirmed by the same group [58]. Also, histological analyses of human herniated discs revealed the presence of infiltrated T cells [59]. Furthermore, in human herniated tissue, lymphocytes were found to be three times more abundant in sequestrated hernias than in extrusions, while no other inflammatory cells were seen in protrusions apart from macrophages [60]. In another study, the inflammatory infiltrate has been characterized by immunostaining in portions of herniated discs which underwent surgery for LDH. None of the 38 samples expressed the immunophenotypic markers of the lymphocyte (CD20, CD45RO, CD4, CD8, TCR), mature monocyte (CD33), or dendritic cell (CD1a, CD80, CD86, S100). However, an abundant infiltration of CD68^+^ cells that lacked CD33 with variable amounts of CD11b, CD11c, and CD40 was observed, likely representing a process of differentiation from monocytes to macrophages [61].

## Conclusions

LDH spontaneous resorption is well documented clinically and in preclinical studies. Spine surgeons are becoming increasingly aware of this phenomenon and many recognize the usefulness of conservative treatment for LDH and advise patients accordingly. Different forms of nonsurgical treatments should be exhausted before considering surgery in acute stages of LDH, unless conservative treatment is contraindicated for reasons such as neurological deficit and intolerable pain despite administration of adequate pain medications.

It is clear that the inflammatory response that occurs associated with LDH is crucial to its spontaneous resorption. Therefore, inflammation in this specific clinical context is a good prognostic indicator and should not be halted. Still, it is exactly an inflammatory response that causes a harmful effect on the adjacent nerve roots, causing pain. The control of the inflammatory reaction in this setting is an important challenge when treating patients with LDH. The combination of knowledge from the biological mechanisms behind LDH resorption and the detailed personalized diagnosis will be the determinant to tailor treatment to each individual patient and may ultimately lead to reduction in costs to the health system.

## Abbreviations

AF: Annulus fibrosus
IVD: Intervertebral disc
LDH: Lumbar disc herniation
MSC: Mesenchymal stem cells
NP: Nucleus pulposus
